# Mapping ethical, legal, & social implications (ELSI) of assisted reproductive technologies

**DOI:** 10.1007/s10815-023-02854-4

**Published:** 2023-06-29

**Authors:** Ido Alon, Zacharie Chebance, Francesco Alessandro Massucci, Theofano Bounartzi, Vardit Ravitsky

**Affiliations:** 1 Department of Development Economics, Autonomous University of Madrid, Madrid, Spain; 2 University of Montreal, Montreal, Canada; 3 Harvard Medical School, Boston, USA; 4 SIRIS Lab, Research Division of SIRIS Academic, Barcelona, Spain; 5 Department of Obstetrics and Gynaecology, Faculty of Medicine, School of Health Sciences, University of Thessaly, Larissa, Greece; 6 Mines Paris - PSL, Paris, France

**Keywords:** Assisted reproductive technologies, Ethical social and legal implications, Mapping, Topic modeling, Geographic distribution of research

## Abstract

**Purpose:**

A significant portion of the research on assisted reproductive technologies explores ethical, legal, and social implications. It has an impact on social perceptions, the evolution of norms of clinical practices, regulations and public funding. This paper reviews and maps the geographical distribution to test the hypothesis of geographical concentration and classifies the output by fields and topics.

**Methods:**

We queried PubMed, Scopus and the Web of Science for documents published between 1999 and 2019, excluding clinical trials and medical case reports. Documents were analyzed according to their titles, abstracts and keywords and were classified to assisted reproductive fields and by Topic Modeling. We analyzed geographic distribution.

**Results:**

Research output increased nearly tenfold. We show a trend towards decentralization of research, although at a slower rate compared with clinical assisted reproduction research. While the U.S. and the U.K.’s share has dropped, North America and Western Europe are still responsible for more than 70%, while China and Japan had limited participation in the global discussion. Fertility preservation and surrogacy have emerged as the most researched categories, while research about genetics was less prominent.

**Conclusions:**

We call to enrich researchers’ perspectives by addressing local issues in ways that are tailored to local cultural values, social and economic contexts, and differently structured healthcare systems. Researchers from wealthy centers should conduct international research, focusing on less explored regions and topics. More research on financial issues and access is required, especially regarding regions with limited public funding.

## Introduction

In the last two decades, the use of Assisted Reproductive Technologies (ART) has been increasing worldwide. The number of yearly performed ART cycles has risen approximately threefold in the United States [14], fourfold in Europe [20, 22], sixfold in Japan [29 ] and by at least tenfold in China [6, 39]. Meanwhile, the volume of research output in the field has expanded even faster, as indicated by the increasing number of high-quality scientific publications [24].

A significant portion of the research on Assisted Reproductive Technology (ART) explores the ethical, legal, and social implications (ELSI) of ART, applying humanities and social science methodologies. This research impacts the way individuals and society perceive ART, the evolution of norms of clinical practices, and the way these technologies are being regulated and funded. [4, 15, 19, 31, 35, 38]. Therefore, influenced by cross-cultural differences and value-based perspectives, ART regulations vary significantly between states and countries [13], which was previously described as legal mosaicism [37]. Moreover, the complexity and weight of regulations increases steadily, due to the growing use of ART, the technical progress, the dissemination of techniques such as gametes cryopreservation, Preimplantation Genetic Testing (PGT), and the emergence of novel possibilities such as Germline Genetic Modification (GGM), among others [4, 5].

Previous reviews analyzed trends in clinical reproductive medical research between 2003 and 2012 [2], and mapped general trends of ART research between 2005 and 2016 [24]. This review, focuses on the ELSI—non-clinical literature encompassing ART, excluding clinical trials, medical case reports and laboratory techniques analyses. Our corpus, which was extracted from PubMed, Web of Science and Scopus, includes 7,714 articles concerning ART applications from humanities and social sciences perspectives.

Geographical concentration of ELSI of ART (i.e., in a limited number of countries or centers) may result in findings that are not representative or comprehensive, since local socio-cultural contexts may shape the framing of, and approach towards research questions and analysis. Admittedly, clinical research in ART can also suffer from being non-inclusive and non-representative. When such research is concentrated in a few countries, or even a limited number of centers of excellence, findings may fail to generate data and clinical approaches that are applicable for diverse populations in other locations and contexts. However, such clinical research in ART could still result in some objective outcomes that can be translated into universally relevant protocols and guidelines. Contrariwise, ELSI literature in ART is shaped in a more profound way by socio-cultural contexts and may thus differ in more significant ways between different societies and circumstances.

This review aims to test the hypothesis of geographical concentration and evaluate its scale. We map the literature published between 1999 and 2019 according to country of corresponding authors, taking into consideration a significant portion of international research (16.3% of the corpus), i.e., those cases in which a corresponding author from one country was conducting research about another country. We report trends and shifts in research focus according to predefined ART fields and by topic modeling, and identify gaps and opportunities for researchers. Furthermore, the classification of the literature into fields and topics will enable to conduct various meta-analyses in future research.

## Methods

### Design

We began by establishing inclusion criteria to select articles dealing with ELSI of ART and exclusion criteria to exclude articles dealing primarily with clinical and medical issues, as described in Table 1.

**Table 1:** Inclusion and exclusion criteria for articles in the review

Inclusion criteria	Exclusion criteria
All articles concerning all sorts of ART and Assisted Insemination (AI) which are dealing either: • Fully or partially with demographic (activity reports and registries, geographical and socioeconomic patterns of usage), ELSI, economic, regulations, anthropological, and political issues. OR • with value-based approaches, attitudes, opinions, expectations, and preferences. OR • with education, knowledge, and empowerment of decision-making among patients and donors. OR • with psychology and quality of life issues, raising ELSI or affecting government policy making and patients’ decision making	Articles which focus on either: • Clinical methods/outcome or technical issues with no relation to ELSI or social sciences. OR • Physicians’ professional approaches, opinions, preferences, education, and knowledge concerning clinical decision-making of technical character. OR • Psychology/quality of life outcomes by way of empirical research with no ELSI or impact on patients’ decision making

### Collection

The corpus was collected from the online databases PubMed, Web of Science (WoS) and Scopus. Since we aimed to analyze titles, abstracts and keywords, we included article that had an abstract in English, regardless of the article’s language.

After running keywords frequency analysis following various preliminary queries, our interdisciplinary team selected three groups of Medical Subject Headings (MeSH)-terms, as shown in Table 2. Group A included ART terms and Group B included terms indicating disciplines within humanities and social sciences. Group C was formed of terms that are typically used by clinical/scientific articles and rarely by writers of ELSI, in order to exclude irrelevant articles. We carefully aimed to find balance between false-positive (inclusion of articles with medical-clinical nature) and false-negative (exclusion of articles concerning social sciences and humanities).

**Table 2:** MeSH-terms and keywords

Group A – Inclusion of ART terms	MeSH-terms (for PubMed) (28)
	Reproductive Techniques, Assisted • Donor Conception • Embryo Transfer • Single Embryo Transfer • Fertility Preservation • Fertilization in Vitro • Mitochondrial Replacement Therapy • Sperm Injections, Intracytoplasmic • Gamete Intrafallopian Transfer • Insemination, Artificial • Insemination, Artificial, Heterologous • Insemination, Artificial, Homologous • Oocyte Donation • Oocyte Retrieval • Posthumous Conception • Embryo Disposition • Sperm Banks • Surrogate Mothers • Preimplantation Diagnosis • Sex Preselection • Gene Editing • Genetic Enhancement • Adult Germline Stem Cells • Germ Cells • Ovum • Oocytes • Embryo Research • Research Embryo Creation
	Keywords (for WoS and Scopus) (38)
	assisted reprod* • assisted procreat* • reproductive techn* • in vitro fertili* • intracytoplasmic sperm injection • In vitro gametogenesis • egg don* • oocyte don* • sperm don* • embryo don* • donor eggs • donor conception • fertility preservation • oocyte cryopreserv* • egg freez* • sperm cryopreserv* • embryo cryopreserv* • oncofertility • leftover embryos • surplus embryos • frozen embryos • preimplantation genetic • reproductive genetic* • germline engineering • germline gene editing • germline gene modification • germline genetic modification • mitochondrial replacement • mitochondrial don* • surrogacy • surrogate mother • gestational carrier • uterus transplantation • artificial insemination • donor insemination • posthumous insemination • posthumous conception • posthumous reproduct*
Group B – Inclusion of terms from disciplines of humanities and social sciences	MeSH-terms (for PubMed) (23)
	Disability Evaluation • Anthropology • Demography • Economics • Forecasting • Policy • Private Sector • Public Sector • Sociology • Work-Life Balance • Education • Stakeholder Participation • History • Knowledge • Philosophy • Religion • Disabled Persons • Vulnerable Populations • Population Characteristics • Health Care Economics and Organizations • Health Services Administration • Health Care Quality, Access, and Evaluation • Attitude
	Keywords (for WoS and Scopus) (28)
	*ethic* • *access* • anonym* • attitude* • perception • *consent* • market* • crossborder • disclos* • eugen* • *identity • justi* • educat* • law* • legis* • legal* • moral* • inmoral • policy* • politic* • govern* • sex select* • touris* • view* • autonom* • desire* • vulnerab* • relatedness
Group C- Exclusion of medical-technical terms	MeSH-terms (for PubMed) (45)
	Ovulation Induction • Neoplasms • Pregnancy Complications • Follicle Stimulating Hormone • Breast Neoplasms • Odds Ratio • Sperm Motility • Prognosis • Semen Analysis • Congenital Abnormalities • Gonadotropin-Releasing Hormone • Ovarian Hyperstimulation Syndrome • Polycystic Ovary Syndrome • Pedigree • Recombinant Proteins • Clomiphene • Zygote • Mice • Reproducibility of Results • Ovarian Follicle • Chorionic Gonadotropin • Sperm Retrieval • Ovarian Neoplasms • Testis • Gonadotropins • Estradiol • Randomized Controlled Trials as Topic • Oligospermia • In Vitro Oocyte Maturation Techniques • Superovulation • Zygote Intrafallopian Transfer • Polar Bodies • Zona Pellucida • Sperm Head • Acrosome • Sperm Tail • Spermatids • Spermatocytes • Spermatogonia • Embryonic Germ Cells • Plants • Case–Control Studies • Botany • Agriculture • Clinical Trials as Topic
	Keywords (for WoS and Scopus) (38)
	ovulation Induction • neoplasms • follicle stimulating hormone • odds ratio • sperm motility • semen analysis • congenital abnormalities • gonadotropin • ovarian hyperstimulation • polycystic ovary • pedigree • recombinant proteins • clomiphene • zygote • ovarian follicle • testis • estradiol • randomized controlled trials • controlled trials • clinical trials • controlled clinical trial • validation study • polar bodies • zona pellucida • sperm head • acrosome • sperm tail • spermatids • spermatocytes • spermatogonia • embryonic germ cells • plants • case control • botany • agriculture • oligospermia • oocyte maturation • superovulation

* = Zero or more characters

We used the PubMed API [41] to query for articles with ‘One MeSH-term from group A’ AND ‘One MeSH-term from group B’ AND ‘Humans (MeSH)’ AND ‘1999–2019’ NOT ‘Any MeSH-term from group C’.

The PubMed query brought up 11,246 results of which 7,003 had a full record of title and abstract in English. Additionally, 159 articles which were queried with no full record from PubMed, were imported from Scopus. In total, 7,162 articles had a full record.

We dropped all abstracts with less than 50 words (259)^1^; removed articles if article type included “Clinical Trial”, Controlled Clinical Trial”, “Randomized Controlled Trial” or “Validation Study” (536); and excluded all journal titles containing the words: "animal", "zoo", "plant", "marine", "poultry", "fish", "insect", "wild", "virus", "bacter", "veterin", "botany", "agricult", "avian", "pest", "bug", "maritime", "aquatic", "xenobiotic" (34). We remained with 6,333 articles and extracted a list of keywords including their frequencies within titles, abstracts and keywords.

Using frequency analysis, we selected three groups of keywords (see Table 2) with similar definition as described above (for the MeSH-terms), and queried the WoS and Scopus APIs for articles of which the title, abstract or keywords had: ‘One term from group A’ AND ‘One term from group B’ AND ‘1999–2019’ NOT ‘Any term from group C’.

We extracted 14,394 and 12,588 articles from WoS and Scopus, respectively. In addition to the 6,333 extracted from PubMed, 33,315 articles were merged from all three databases. Following the removal of duplicates of titles, abstract and DOI, 17,247 articles remained, of which 14,283 had available title and abstract in English. We repeated the cleaning methods previously applied on the PubMed query (explained above), removed 154 articles due to short abstract (less than 50 words), and 99 due to journal names including the words “animal”, “Zoo” and others (see above). 14,030 articles remained.

### Manual cleaning

Two researchers cleaned up the database over the course of six months by analyzing the articles’ titles and abstracts with an emphasis on the following rejection criteria (derived from Table 1): Articles dealing exclusively with 1. Animal research. 2. Treatment outcome, unless measured in terms of population and socio-economic characteristics (i.e., national registries). 3. Clinical policies on a local level (in contrast to national/regional policies). 4. Clinical outcome and performance of technologies and protocols. 5. Processes within hospitals and clinics. 6. Prenatal testing and selection. 7. Therapeutics (non-ART) uses of stem-cell research. 8. Clinical trials or reports in psychiatry/psychology with no ELSI or impact on patients’ decision making.

We removed 6,316 articles that did not meet our criteria, leaving a database of 7,714 relevant articles of full record. Among these 7,714 articles, 1,184 were non-English articles with title and abstract in English, allowing for analysis and classification. These non-English articles made up 15.34% of the database.

### Classification

The abstracts were processed by merging the title and abstract into one string (“code”). We harmonized the “codes” to British English, tokenized^2^ each sentence into a list of words, removed punctuations and unnecessary characters and replaced upper-cases with lower-cases. Stop words (‘a’, ‘the’, ‘is’, ‘are’ etc.) were removed^3^ and the text was lemmatized (inflectional endings were removed, leaving the root words).^4^ We formed a list of terms to be replaced with acronyms or abbreviations in order to unify the text and allowing us to identify repetitions. For examples: ‘in vitro fertilization’ was replaced with ‘ivf’, ‘oocytes cryopreservation’ and ‘eggs cryopreservation’ with ‘eggscryopreservation’.

Next, we extracted a list of terms by the frequency of “codes” in which they appear and divided the most frequent technical-medical terms into ten ART fields (see Appendix 1): 1. Egg Donation; 2. Sperm Donation; 3. Embryo Donation; 4. Surrogacy; 5. Fertility Preservation; 6. Stem-cell Research; 7. PGT; 8. Genetic Modification; 9. MRT; 10. Assisted Insemination. An article was assigned into an ART field (or several) if one term, associated to that field, was found in its title, abstract or keywords. The articles which included none of these terms were assigned by two researchers reading their titles and abstracts. The remaining articles (2,267/7,714) were appointed as “General”.

Subsequently, for each publication, all obtainable metadata was extracted from WoS, Scopus and PubMed, merged and unified under one template. Finally, we cleaned the abstracts from all those technical-medical terms that were used to define the 10 ART fields and then: 1. Uploaded the database to the VOSviewer software tool for constructing and visualizing bibliometric networks. 2. Applied Topic Modeling (TM) via Latent Dirichlet Allocation (LDA) [9. , 32. ] to all “codes”. To do so, we've built a corpus dictionary using gensim^5^ open-source library.^6^ The LDA method assumes that the observed distribution of words in a textual corpus is determined by a statistical model that fixes both a word-topic and an article-topic association [24].

**Fig. 1:**
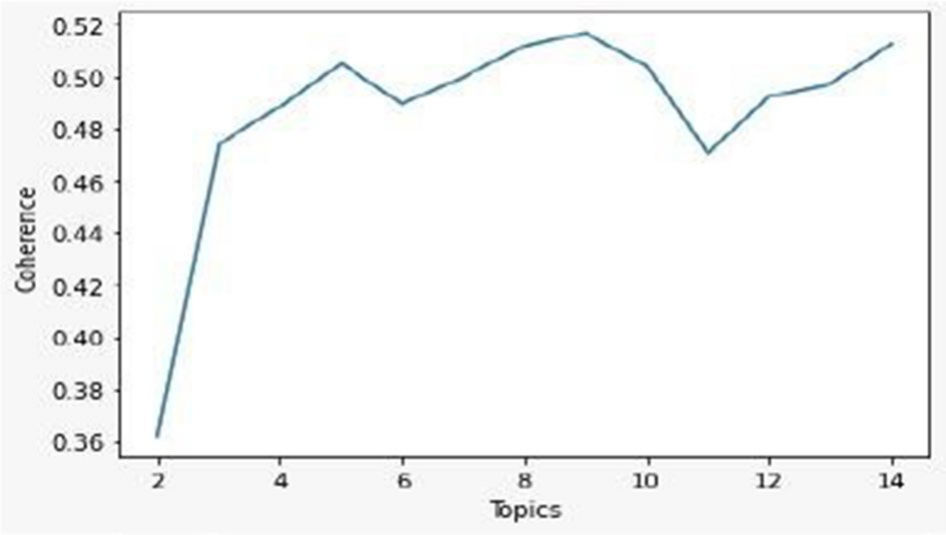
Coherence Score as a function of the number of topics

We defined the number of topics by computing the coherence score as a function of the number of topics^7^ and by assessing the results in relation to the VOSviewer analysis. The results of the LDA algorithm consist in a list of topics, and in the weighted relations (0 to 1) between each article and each topic. Every topic is a list of characterizing words, and each article may be connected to more than one single topic. Thus, as seen in Fig. 1, at least 5 topics should be identified. According to our analysis, we defined 6 topics as the most accurate solution. Each article was associated to those topics by considering a weighted relation of more than 0.333334 so that each article could be associated to not more than two topics. As a result, only 161 articles remained unassociated.

### Analysis

In addition to the VOSviewer tool, we conducted an analysis using Microsoft Excel Spreadsheet based on year of publication, countries of corresponding authors and countries mentioned in codes as an indicator for the focus of the article. For the last, we searched the codes (abstracts, titles and keywords) using a list of countries, cities (with no duplications), and nationalities.^8^ART Fields, topics identified by the TM, regions,^9^ and income level.^10^ We used the IBM SPSS Statistics tool to test for Spearman’s correlations between the ART fields and the topics (Appendix 2). Those correlations were used for two purposes. First, to group ART fields in order to simplify the results, i.e., [Egg (C1) and Sperm donation (C2)], and [Stem-cell Research (C6) PGT (C7) and Genetic Modification (C9)]. Second, to verify a relationship between ART fields and topics.

## Results

Between 1999 and 2019, the global research output in the field grew about 12% annually. Therefore, the annual output increased almost tenfold (× 9.75), from 72 publications in 1999 to 702 in 2019. We begin by analyzing research areas on two axes, the first includes eleven ART fields (10 technical fields plus General) and six Topics (clusters) identified by both TM and the VOSviewer analysis. Additionally, we present a geographical analysis of the database.

### Areas of research

**Fig. 2:**
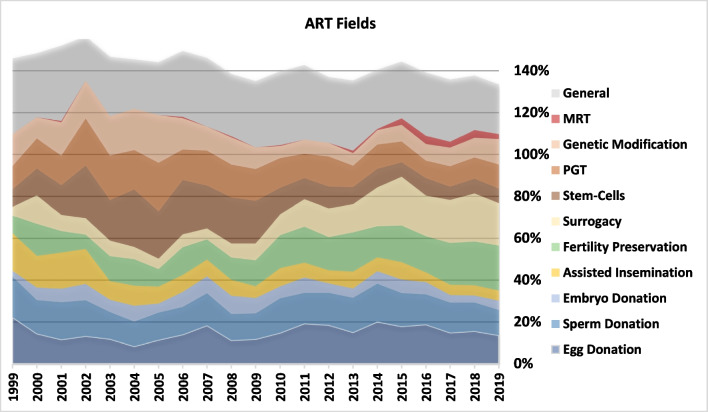
ART fields

**Fig. 3:**
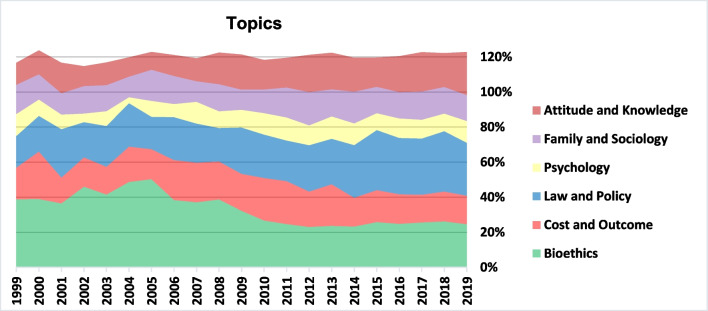
Topics

Some articles were assigned, to more than a single ART field or a single topic. For this reason, in Figs. 2 and 3 (as well as, Figs. 5, 8 and 9), the total sums-up to more than 100%. The three ‘Donation’ fields had a variable trend during the two decades which ended in an overall decrease from 44% (1999) to 30% (2019) of the total. The share of ‘Assisted insemination’ has decreased steadily while both ‘Fertility Preservation’ and ‘Surrogacy’ are the two emerging fields throughout this period, particularly in the second decade. In the last three years each of these two fields was engaged by more than 20% of the literature. Furthermore, ‘Stem-cells’, ‘PGT’ and ‘Genetic Modification’ were the most explored fields at the beginning of the previous decade, occupying 68% of all publications in 2005. During the second decade, interest in the genetics of ART has moderated while the field of ‘MRT’ has emerged.

Based on Spearman correlations (Appendix 2) we grouped the fields ‘Egg Donation’ and ‘Sperm Donation’ (into ‘Egg & Sperm Donation’), as well as, the fields Stem-cell research, PGT and Genetic Modification (into ‘Genetics’), in order to simplify result presentation in the forthcoming analysis.

**Fig. 4:**
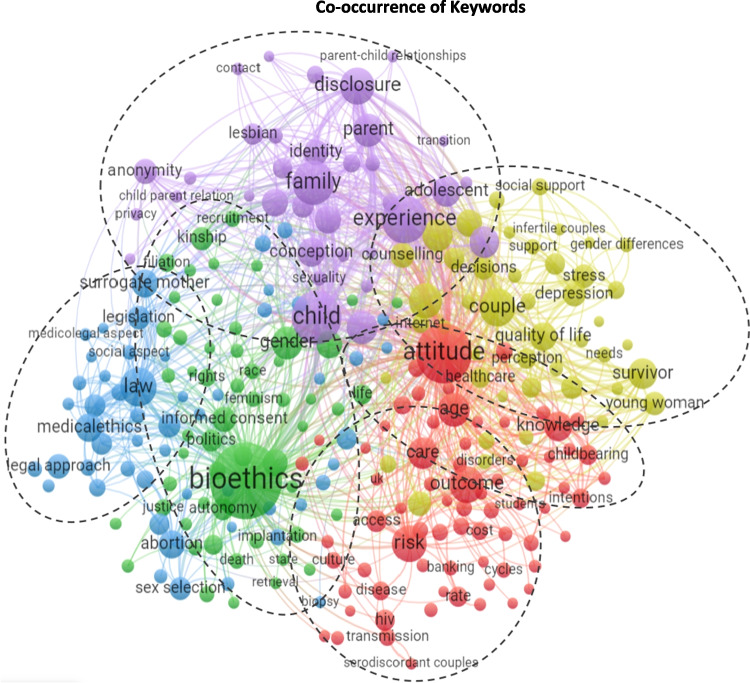
Co-occurrence of keywords

We used the VOSviewer software tool to find co-occurrence links between terms.^11^ In Fig. 4, the size of the label and the frame of an item is determined by the weight (number of occurrences) of the item. Thicker Lines between items represent stronger links [30]. The colors represent 5 clusters. We invite the reader to consult the interactive version of this figure, available online at: https://app.vosviewer.com/?json=https://drive.google.com/uc?id=1iQXv7oEQ6RE7-wz-578G00ULsLfkL3ao

Additionally, the TM raised 6 topics which are characterized by lists of words of which we displayed the first 10 for each topic, as seen in Table 3 (full output in Appendix 3). We assigned these topics to the clusters identified by the VOSviewer analysis (The Red was the largest cluster and was assigned with two topics):

**Table 3:** Topic modeling

	Topic	Keywords
1	Bioethics (Green)	Ethical, Genetic, Moral, Medical, Legal, Ethic, Scientific, Principle, Development, Medicine
2	Cost and Outcome (Bottom Red)	Patient, Transfer, Rate, Cycle, Clinical, Procedure, Risk, Clinic, Cost, Ethical
3	Law and Policy (Blue)	Law, Legal, Country, Social, Policy, Regulation, Legislation, Medical, Work, Analysis
4	Psychology (Yellow)	Couple, Psychological, Infertile, Counselling, Factor, Patient, Participant, Social, Relationship, Interview
5	Family and Sociology (Purple)	Child, Parent, Family, Couple, Mother, Lesbian, Conceive, Genetic, Sex, Relationship
6	Attitudes and Knowledge (Top Red)	Patient, Age, Couple, Risk, Attitude, Knowledge, Medical, Genetic, Young, Participant

We may clearly see a decrease in the share of topic ‘Bioethics’ with increases in the shares of both ‘Law and Policy’ and ‘Attitudes and Knowledge’. The share of ‘Psychology’ decreased in the first decade but recovered during the second. We should remind that, due to the database selection, ‘Psychology’ includes only articles with ELSI or impact on patients’ decision making and not all psychological research concerning ART.

**Fig. 5:**
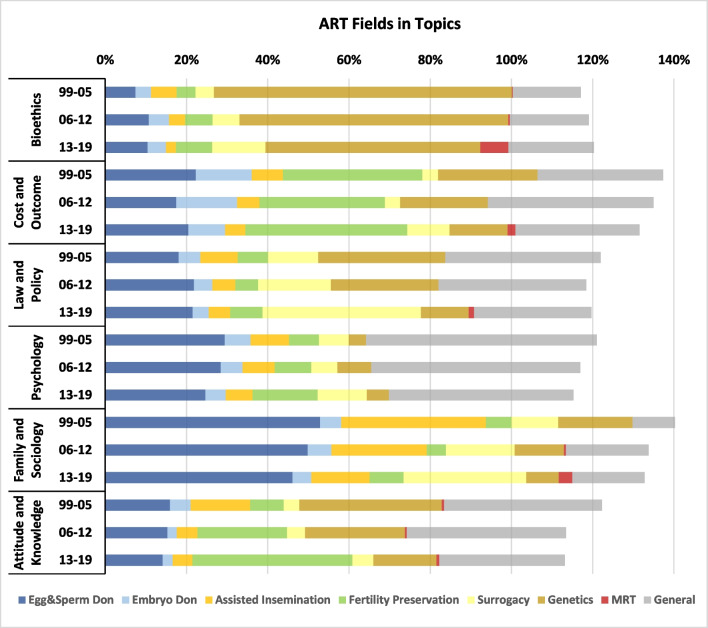
ART fields in topics

As seen on Fig. 5 and according to Spearman’s correlations presented in Appendix 2, we note that ‘Bioethics’ is strongly focused on the ‘Genetics’ field, though this focus has decreased with time, from 73% in the first period to around 53% in the last. The field ‘Egg & Sperm Donation’ has gained share under the topics ‘Bioethics’ and ‘Law and Policy’. It was nevertheless the focus of More than 45% of the topic ‘Family and Sociology’, while a relatively high but decreasing share of this topic dealt with ‘Assisted Insemination’. Concerning the two emerging fields, 'Fertility Preservation' was most strongly covered under the topics 'Cost and Outcome’ and 'Attitudes and Knowledge', while ‘Surrogacy’ was highly under the focus of the topics ‘Law and Policy’ and ‘Family and Sociology’. The decreasing total percentages indicates that research is becoming more specialized, i.e., for all topics except for ‘Bioethics’, with time, the average number of fields related to a single article is decreasing. The topics ‘Cost and Outcome’ and ‘Family and Sociology’ are more multi-field than the others.

### Geographic analysis

**Fig. 6:**
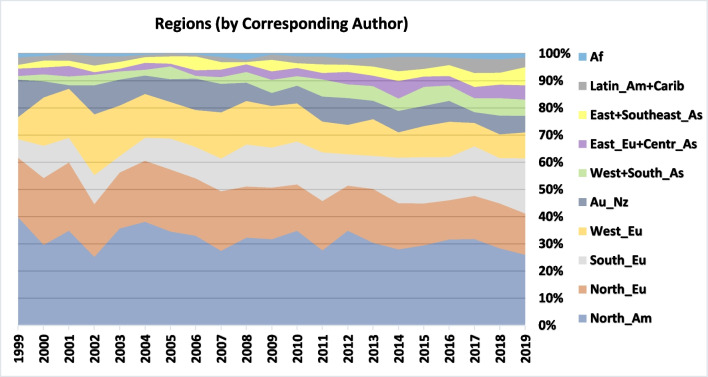
Regions (by Corresponding Author)

**Fig. 7:**
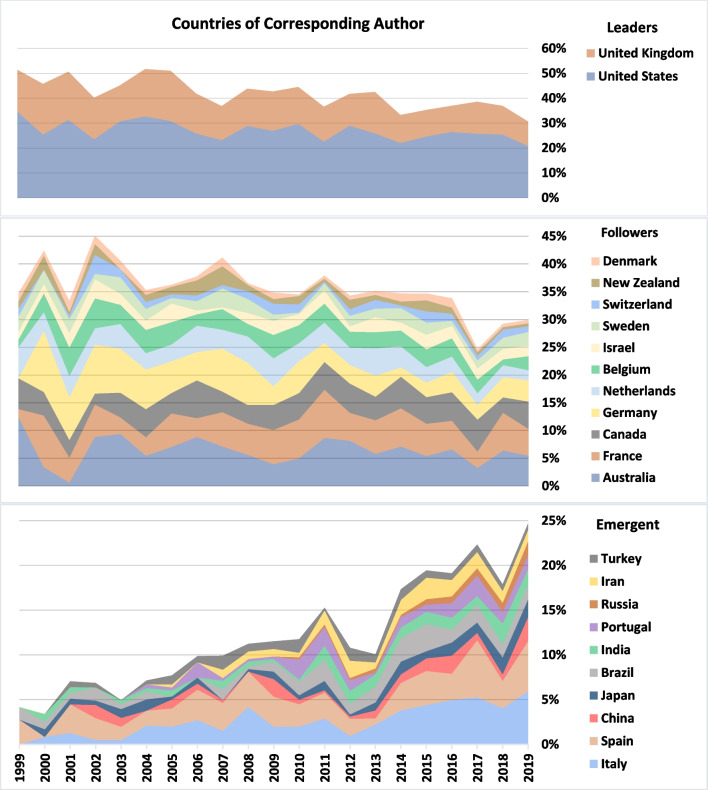
Countries of corresponding author. The leaders and followers are ordered by the total publications of 1999–2019 while the emergent countries are ordered by the publications of 2019

Figures 6 and 7 present the shares of publications per regions and leading countries^12^ respectively. In 1999, the five leading regions Northern America, Northern, Southern and Western Europe, as well as Australia-New Zealand were responsible for 90% of the world publications (according to corresponding authors). Nevertheless, in 2019 the share of the five leading regions decreased to less than 76.5%. The Northern American share decreased from 40 to 26%, while Southern Europe’s share increased from 7 to 20% and this region became the second most engaged.

The two leaders, the US and the UK, fell from 51% in 1999 to 31% in 2019, although still holding a significant share of global publications. In the middle part of Fig. 7, we may notice that among the eleven followers, the shares of Australia, Canada, New Zealand, the Netherlands, Germany and Denmark, all decreased significantly, while the others have been relatively stable.

On the bottom part of Fig. 7, we observe the emergence of Spain (the fourth largest ART user in the world) and Italy, as well as the awakening of two world’s leaders in terms of ART cycles, China and Japan, which during the last decade increasingly contributed to the global ART literature.

Of the 7,714 articles included in this review, 3,743 (48.5%) were identified as dealing with specific countries, while 1,260 (16.3%) were international research, in which the corresponding author was based in one country while conducting research about another.

Table 4 demonstrates the 20 leading countries in international research alongside the 23 most popular research subjects. All of the leading countries in the field of ELSI of ART were also engaged in international research, despite their research being largely an exchange between the leaders and the followers, with the exception of India, that attracted a large number of foreign researchers (121 articles), particularly in the field of surrogacy (85 articles).

**Table 4:**
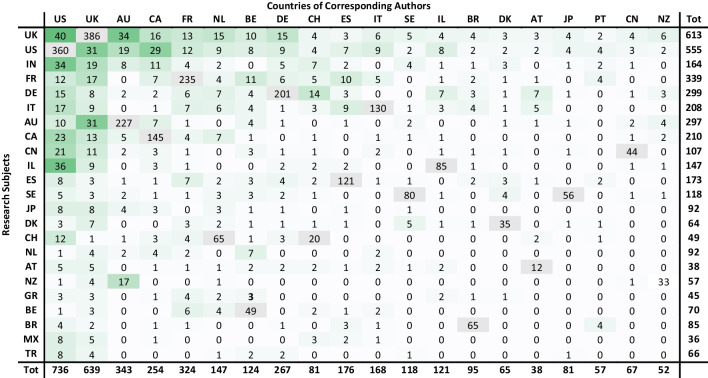
Matrix of international research within the ELSI of ART

Wealthy nations (according to the World Bank’s level of income) were much more engaged in research about ELSI of ART. Hence, 89% of all publications had a corresponding author from a high-income country, only 8% from a high-middle income country, and 3% from a low-middle income country (there were only 14 publications from low-income countries throughout the entire period). High-income countries had more focus on ‘Egg & Sperm Donation’ and on ‘Genetics’ while high-middle and low-middle income countries had more focus on ‘Surrogacy’. The most engaged among the high-middle and low-middle income countries were Brazil, Iran, India, Turkey, China, South Africa, Russia, Romania, Nigeria, and Mexico.

**Fig. 8:**
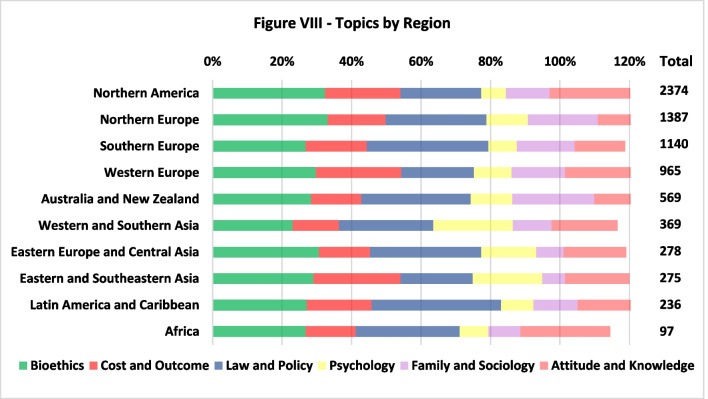
Topics by region

There were no remarkable specializations of certain regions in certain topics, as seen in Fig. 8. For all topics, the US leads soundly, followed by the UK. The following countries in all topics are Australia (with the exception of ‘Cost and Outcome’) and France (with the exception of ‘Psychology’). The relatively high share of the topic ‘Psychology’ among Western and Southern Asian publications could be explained by the attention-grabbing focus of Iran in this topic (53 out of its 87 publications), which makes it the fourth most publishing country in this topic during those two decades. Moreover, for all topics exccept ‘Psychology’ (66%), the 10 most publishing countries are responsible for 70–80% of the global publications.

**Fig. 9:**
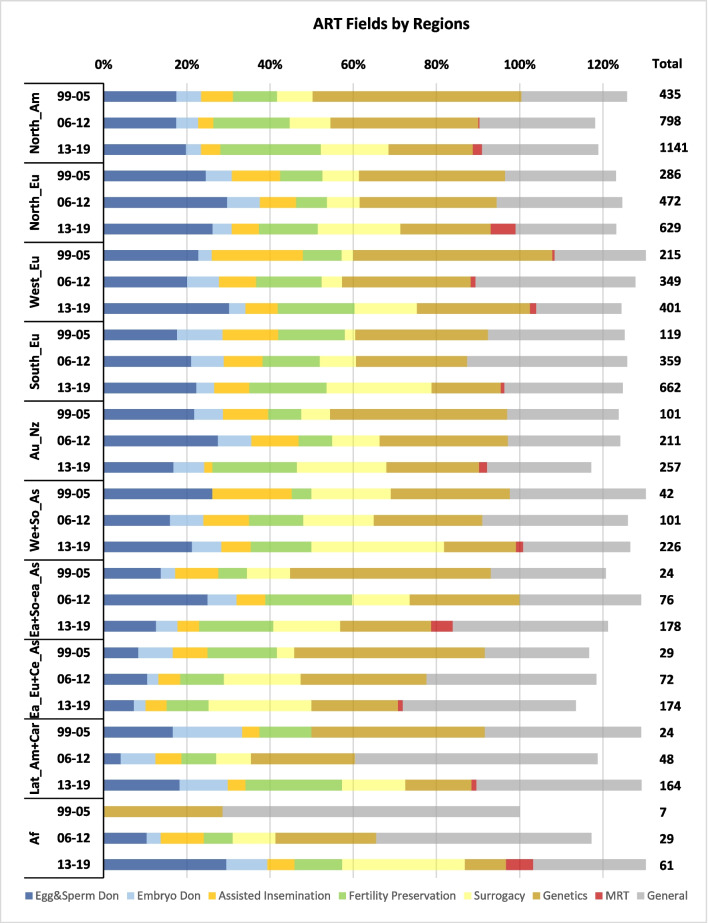
ART fields by regions

There were some significant trends in ART fields (Table 5 and Fig. 9). The US was the leader in almost all fields during the two decades and was always followed by the UK, that led only in publications concerning ‘MRT’ (with 34% of the total). In this emerging field, Canada was third, and the ten leading countries were responsible for 82% of the global publications. In the field of ‘Egg & Sperm Donation’, Italy emerged strongly, reaching the top five at the end of the period. In the minor field ‘Embryo donation’, German authors were dominant, publishing 5% of the global amount. Iran was also strong in this field and was 6^th^ with 17 publications (14 of them in the second decade). Research concerning ‘Surrogacy’ was the most decentralized as “only” 69% were published by the top 10 countries. In this field Spain and Italy emerged to become major publishers. Under the ‘Genetics’ fields, particularly concerning PGT, Germany was the third strongest publisher. However, its engagement was felt more at the first decade, whereas Australia was stronger at the second decade. In all the three fields, ‘Egg &S perm Donation’, ‘Fertility Preservation’ and ‘Genetics’, 75% of global publications were published by the top 10 countries.

**Table 5:** Countries by ART fields

Egg & Sperm Don										
1999–2005	US	UK	BEL	DEU	AUS	FRA	ISR	NED	NZL	SWE
	28.1%	23.9%	7.7%	6.2%	5.4%	5.00%	3.8%	3.5%	3.1%	1.9%
2006–2012	US	UK	FRA	AUS	DEU	CAN	NZL	BEL	SWE	ESP
	21.7%	20.6%	9.9%	6.9%	5.8%	4.3%	3.9%	3.7%	3.0%	2.8%
2013–2019	US	UK	FRA	BEL	ITA	AUS	CAN	NED	ESP	DEU
	22.5%	14.9%	7.4%	6.2%	4.6%	4.5%	4.4%	3.3%	3.3%	3.3%
Embryo Donation										
1999–2019	US	UK	AUS	FRA	DEU	IRN	ITA	BEL	SWE	CAN
	22.0%	13.8%	7.5%	6.8%	5.0%	3.9%	3.2%	3.2%	3.2%	2.9%
Assisted Insemination										
1999–2019	US	UK	FRA	DEU	ITA	NED	BEL	AUS	NZL	ISR
	17.3%	16.6%	9.1%	9.1%	4.5%	4.4%	4.4%	3.7%	3.3%	3.0%
Fertility Preservation										
1999–2012	US	UK	BEL	FRA	AUS	DEU	NED	CAN	ITA	ESP
	36.1%	11.5%	6.1%	5.6%	5.0%	4.4%	4.4%	4.2%	3.8%	2.5%
2013–2019	US	UK	AUS	FRA	CAN	ITA	DEU	ESP	BRA	SWE
	31.9%	9.4%	6.5%	6.1%	5.2%	4.3%	3.8%	2.7%	2.6%	2.0%
Surrogacy										
1999–2012	US	UK	AUS	FRA	CAN	DEU	IND	NZL	NED	GRC
	30.1%	16.9%	6.8%	5.8%	5.2%	4.0%	3.7%	2.7%	2.5%	2.5%
2013–2019	US	UK	ITA	ESP	AUS	CAN	FRA	IND	DEU	SWE
	18.7%	10.5%	6.9%	6.2%	6.1%	4.8%	4.8%	3.9%	3.3%	2.8%
Genetics										
1999–2005	US	UK	DEU	AUS	FRA	CAN	NED	BEL	ESP	SWE
	35.9%	15.6%	10.5%	7.2%	3.8%	3.6%	2.9%	2.9%	2.0%	1.6%
2006–2012	US	UK	AUS	DEU	CAN	ESP	FRA	NED	ITA	BEL
	29.3%	15.6%	7.2%	6.9%	6.1%	4.0%	3.4%	3.1%	2.1%	1.7%
2013–2019	US	UK	AUS	DEU	NED	ITA	FRA	CAN	ESP	BEL
	25.7%	13.5%	5.9%	4.8%	4.4%	3.9%	3.7%	3.5%	2.9%	2.5%
MRT										
2001–2019	UK	US	CAN	NED	AUS	ZAF	DEU	JPN	SGP	CHN
	34.3%	18.5%	6.5%	5.6%	4.6%	3.7%	2. 8%	2.8%	1.9%	1.9%

To summarize (Fig. 9), Northern, Western and southern Europe were slightly more engaged in the research about ‘Egg & Sperm Donation’, while Northern America, as well as Australia and New Zealand were more focused on ‘Genetics’ and ‘Fertility Preservation’. The regions which focused more on Surrogacy were Southern Europe, Western-Southern Asia, and Eastern Europe and Central Asia.

## Discussion

We conducted this review to map out the geographical distribution of ELSI of ART research output published between 1999 and 2019, according to country of corresponding authors. We also sought to explore research themes in order to identify concentration of research attention and gaps. Geographic/regional and topical centralization of ELSI studies on ART produces research gaps. Such studies are intended, amongst other things, to inform state and professional regulations that largely differ between nations due to local legal, social and economic contexts, cultural values, and diverse structures of healthcare systems. Diversity of research locations and topics is important to ensure attention is given to all relevant ELSI themes in various cultural and socio-economic contexts. Lack of diversity creates blind spots that are problematic for patients, families, communities, clinicians, and decision-makers.

In these 21 years, ELSI of ART research output increased nearly tenfold, following an average yearly growth rate of 12%. This growth was much higher compared with that of the entire literature on ART (including all ELSI and clinical/scientific articles), which had an overall increase of only 20% between 2005 and 2015, i.e., an average yearly growth rate of merely 1.7% [24]. Growth in the output of ELSI of ART was also much higher than the 4% annual growth rate of global scientific output between 2008 and 2018, as reported by the US National Center for Science and Engineering Statistics [42].

### The need for de-centralized and diverse ELSI research on ART

Overall, our findings show a clear trend towards decentralization of ELSI of ART research, but they also show that much work remains to be done in terms of further diversification of research regions and themes. The Global scientific output has been centralized for many years within the U.S. and the U.K. as dominant centers of academic research. Our early hypothesis was thus that publications on ELSI of ART would be centralized in a limited number of countries. However, scientific research has become more globalized in recent years, with the share of the U.S. and the U.K. declining. In particular, China’s contribution to research output has grown remarkably, both regarding general science and in relation to ART research. The previous centralized landscape has been replaced by a more diverse academic world, centered in North America, Europe, and Asia–Pacific [18, 40, 42].

Our review shows that research on ELSI of ART has indeed followed this trend over the past two decades, but to a considerably lesser extent compared with scientific research or clinical ART research. In ELSI of ART publications, while the U.S. and the U.K.’s share has dropped from more than 50% in 1999 to around 30% in 2019, North America and Western Europe (including north and south) are still responsible for more than 70% of the output, and adding Australia and New Zealand increases the number to 80% (Fig. 6). While international research helped mitigate the concentration of research in a limited number of countries, a large part of it was conducted across countries in the leading regions (Table 4).

These figures may raise concerns if we consider that China and Japan are currently the world leaders in annual ART cycles, and that China alone performs more cycles than the European Union, the U.K. and Russia combined [6, 21, 39]. Indeed, regarding clinical ART research output, China has become a world leader in recent years, third after the U.S. and the U.K. [24]. However, regarding research on ELSI of ART, Eastern and Southeastern Asia were responsible for merely 4–6% of the global output in the end of the studied period, between 2016 and 2019, still far behind North America and the European regions.

There are several possible explanations for this discrepancy. One might be that language barriers are reducing research output in this area. However, other countries that face language barriers, such as Spain (a world leader in ART) and Italy, have been increasingly contributing to ELSI of ART, showing that such barriers can be overcome. Further, it is possible that some or much of the work in this area is published in local languages, such as Mandarin or Japanese, a question that can be empirically assessed by future research. In our corpus, out of the 1,184 non-English articles (with English abstracts) only 11 articles were written in Mandarin and 12 in Japanese, and most (88%) were in German, Spanish, French, Italian and Portuguese.

Another possible explanation might be cultural differences. ELSI issues might be framed differently in countries with non-Western value systems, affecting the type and quantity of research in this area. Cultural framing may even influence the perception or categorization of certain aspects of ART as ‘issues’ that ought to be researched. What may be seen in one socio-cultural context as a problem that needs to be addressed, might be seen in another as a mere social fact that does not raise concerns worthy of investigation.

In China, many of the ART techniques often discussed by western literature are restricted by law. For example, surrogacy, egg donations (except for sharing between patients), embryo donation (except for research purposes), and fertility preservation (except for married couples and for medical reasons) are prohibited. Other practices, such as sperm donation, are marginalized by social norms. In general, ART is allowed only for “healthy”, officially married couples, and the eligibility for LGBTQ individuals is barely even discussed [1, 27, 33. ]. In Japan, many of these issues were, at least until recently, unregulated and marginalized. Overall, ART practices usually associated wth ELSI research are much less common in Japan, compared with other ART leading countries [28]. Thus, different context and framings may have an impact on the quantity of research that is conducted and published in various regions.

In light of differences in cultural values and hence in regulations between nations, cross-border reproductive care provides opportunities to overcome legal barriers for those who can afford it, but can create inequalities, tensions and frustration [26], and increase risks to women and children involved. Consequently, there are often attempts to discuss ART regulations at the international level, particularly concerning exceedingly controversial issues such as genetic modification and surrogacy. Often, ELSI studies are elaborated as an ongoing international discussion between scholars and experts around the globe. The key to a successful global environment of regulatory collaboration, at least regarding some critical ART applications, is the production of more comprehensive and diverse research. We call to enrich researchers’ perspectives with diverse local expertise, addressing local issues in ways that are tailored to local cultural values, social and economic contexts, and differently structured healthcare systems. This need for diverse research on ELSI of ART is particularly salient regarding countries and regions with a high use of ART.

### Trends in research fields and topics

Some of the trends our findings have identified regarding ART fields and research topics are unsurprising. In most countries with developed ART services, egg, sperm and embryo donations concern a very large share of ART cycles and raise frequent socio-ethical tensions. The share of these fields in the literature fluctuated throughout the last two decades and despite declining by 2019, it is hard to determine a downtrend (Fig. 2). In European regions, where both an aging population and delayed childbearing are particularly prominent, we observed a significant and mostly increasing proportion of ELSI research about egg, sperm, and embryo donations (Fig. 9). The heightened interest in these areas is probably closely tied to the socio-ethical questions they provoke, such as the definition of parenthood, parental rights and responsibilities, as well as the rights of children to be informed of their genetic origins. Consequently, these matters have led to a diverse range of regulations and clinical norms across the European continent. It's noteworthy that the largest shares of ELSI research on egg and sperm donations occur in Northern and Western Europe, regions where the most stringent legal restrictions are in place. The emergence of research on ELSI of fertility preservation and MRT is also understandable, as both technologies matured and became practical during the study period.

Some specific findings can also be explained by considering local factors. For example, the UK led in publications concerning MRT (34% of the total papers on this topic), possibly since it was the first country in the world to regulate the practice following a national consultation [16]. In this emerging field, Canada was third, which can be explained by the opposite reason – MRT is legally banned there and conducting it would constitute a federal criminal offence, which led to a discussion of this barrier for Canadians [16]. In the field ‘Egg & Sperm Donation’, Italy emerged as a strong contributor (2013–2019), possibly effected by the 2014 rule of the Italian Constitutional Court which overturned the ban on gamete donation [11]. The focus on ELSI of fertility preservation in the U.S. could be driven by the advent of novel techniques for cancer patients, notably children, and the vigorous socio-ethical debate surrounding social egg freezing. This trend, largely unsupported by evidence, is propelled by commercial incentives, media coverage, and corporate benefits, yet is accompanied by a dearth of adequate information. The societal implications of advanced maternal age are being addressed, often controversially, by this expensive, unproven method, which tends to shift the burden onto individual women [7, 34 ].

In the field of ‘Embryo donation’, German authors were dominant, which can possibly be explained by some unique German values, attaching the status of person to the human embryo [12] and making the option of donating embryos for reproduction preferable to other options such as destruction or donation for research and training. Similar cultural explanations can clarify why Germany was the third strongest publisher on the PGT, and why its engagement was felt more at the first decade (1999–2009), a time during which PGT was legally banned there for historical and cultural reasons [10] and during which much bioethics debate surrounded this ban.

As noted, research on ‘Surrogacy’ was the most decentralized (69% of papers published by the top 10 countries), with Spain and Italy emerging as major publishers, in addition to Austria (Table 5). This can possibly be explained by the ban on surrogacy in these countries raising discussions on this matter. The focus on ELSI of surrogacy in Canada can be attributed to the federal legislation prohibiting surrogate payment. This sparked a prolonged debate on the permissibility of specific reimbursements and compensations, which was finally resolved after many years, filling a widely criticized policy void [8]. In France, the Bioethics Act of 1994 placed strict limitations on surrogacy [23] resulting in substantial ELSI inquiries. This act, effectively stifling surrogacy agreements, resulted in complex legal predicaments for couples seeking surrogacy abroad. These included complications in acknowledging parentage and securing citizenship for their children, with some children only gaining recognition as citizens upon reaching adulthood.

Moreover, a large share (31%) of international research on surrogacy were focused on India (and 5% on Thailand). This could be partially explained by the ethical tensions surrounding the use of surrogates from low-income countries, especially by couples from high-income countries [36] and especially when access to surrogacy is limited in the commissioning couple’s country of origins.

However, the decline in the share of ELSI research on stem cell research, PGT and genetic modification is quite unexpected, considering the improvement in these technologies throughout the study period, the increased use of preimplantation genetic screening, and the breakthroughs in CRISPR technologies since 2012. Certainly, a decrease in the share of the ‘Genetics’ field is not a decline in absolute terms, i.e., the number of articles addressing ‘Genetics’ increased in absolute terms over the last two decades, but less than other fields. Yet, with the frequent addition of PGT (mainly PGT-A) to ART [14], the advancement in germline genetic modification, one would expect an even faster growth of the discussion of socio-ethical tensions concerning these technologies. This decline could be attributed to several factors. Between 1999 and 2005, breakthroughs such as cloning and the completion of the human genome project stimulated significant attention. However, after 2005, the excitement around these technologies may have begun to stabilized, and the establishment of regulatory frameworks could have prompted a shift in research focus. Additionally, as researchers became more aware of recurring ethical and procedural considerations, they might have refrained from duplicating studies. It is also important to remember that influential contributions in these areas often come from grey literature, such as reports from leading organizations, not just from peer-reviewed academic papers.

Observing the topics raised by LDA (Fig. 4), due to the correlation between the topic Bioethics and the fields Stem-Cells, PGT and Genetic Modification, we may associate the drop in the portion of bioethics with the decline in the portion of these genetic fields (Appendix 2). We also identify a significant increase in the portion of ‘Attitudes and Knowledge’ which could be related to the growing interest in public understanding of both infertility and ART. ‘Law and Policy’, which include many comparative studies, became a more popular type of research throughout the period in all regions. Conversely, the share of ‘Cost and Outcome’ declined towards the end of the period, and we would have expected more publications of this topic, mainly in less wealthy regions where access to ART remains a critical ethical issue. Considering that even in countries with a fairly robust public health system, ART remains at least in part a private service [3], and that in many places around the world a large share of the population struggles to fund and access ART, we would suggest that there is a challenging gap in the literature concerning cost and the burden of ART funding on families. This research direction would be particularly interesting in the U.S., China, and in LMICs. It is also important to explore barriers to access in light of local cultural norms related to reproduction and the stigma of infertility, which still constitutes a significant psycho-social burden in several regions.

### Study limitations and future research directions

Our analysis is not exempt from some methodological limitations. First, our selection and cleaning process had a certain level of subjectivity. The selection criteria were complex and included terms selected by the authors. During the second search cycle focusing on the Web of Science and Scopus, we grouped keywords based on frequency analysis of the articles collected from PubMed. While the initial group addressing Assisted Reproductive Technologies (ART) was comprehensive, the subsequent group intended to encapsulate ELSI research might have benefited from an expansion to include additional terms such as 'anthropology,' 'kinship,' 'cultural beliefs,' and 'social structure.' The omission of these terms potentially explains why anthropology as a topic was not captured in our resulting topic modeling. In our cleaning process, many articles were “manually” removed by analyzing their abstracts, and both false-positives and false-negatives were possible. Second, to facilitate our analysis, we limited our selection to articles with titles and abstracts available in English, therefore, inadvertently excluded some articles published in local or regional journals. Third, in the classification process, the ART fields were defined according to keywords selection, while the occurrence of one term in the abstract indicated an affiliation to an ART field which could lead to some mistakes. Fourth, associating an article to a country based solely on the affiliation of the corresponding author reflects the funding source. Alternatively, focusing on the research topic, as well as co-authors, could indicate that other countries were involved or studied, as shown in a preliminary analysis of international research (Table 4). We further explore this in another article.

Finally, topic modeling by LDA has some limitations [25]. The algorithm assumes a certain probabilistic distribution behind the word/article association, which may not hold in reality. Moreover, there is no "standard" or objective way of fixing the number of topics, which remains a free parameter, adjusted in accordance with different metrics. Consequently, due to the number of topics one eventually selects, some smaller topics may remain hidden. It is also important to remind that in topic modeling the categorization of articles into topics is determined solely by the specific terminology found within each article. Despite these limitations, our findings are based on a comprehensive corpus collected from three major databases, a relatively strong LDA and an additional method of categorization.

In terms of developing our mapping strategies, it would be appealing to analyze international research by identifying studies that were conducted by a corresponding author of one country, but were in fact co-authored by researchers from other countries or were dealing with a different country as a topic. In this paper, we present a preliminary analysis of international research in the ELSI of ART. Our database allows to identify the focus of international research on specific fields and topics, as well as trends over time. Moreover, the division of the database into ART fields and topics would enable various meta-analyses by extracting a single ART field (or topic) and presenting its distributions of study designs, key issues, research questions and outcomes, according to geographic location and timeline.

## Conclusion

In this paper we mapped the ELSI of ART literature over 21 years. We have shown geographic centralization in research, based on corresponding author, which reflects an unequal distribution of research across nations. While it is hard to expect a redistribution of research funds, and although our search parameters might not have captured all pertinent international research in the field, it is required and realistic to encourage researchers from wealthy academic centers to conduct more international research and focus on less wealthy or less explored regions and topics. We conclude that the literature on the ELSI of ART is mainly produced by North America, Western Europe and Australia, although it has been slowly decentralized. In the last few years, the leading emerging fields in ELSI of ART researchers have focused on were surrogacy and fertility preservation, with a strong focus on law and policy. Research about patients’ attitudes and knowledge has been rising, while more research on financial issues and access to treatment is required, especially regarding regions with limited or incomplete public funding of ART.

## Data Availability

The data that support the findings of this study are available on request from the corresponding author, [IA].

## Appendix 1

Table 6

**Table 6:** Words and terms for division into ART fields

Axis A	
Egg Donation	biobanking of eggs and embryo • buying and selling gametes embryos • child donor • donated egg • donated gamete • donated oocyte • donating eggs • donor art • donor child • donor conceived offspring • donor conception • donor egg • donor gamet • donor of gamet • donor of sperm or egg • donor offspring • donor oocyte • donor registry • donor sibling • egg bank • egg don • egg shar • eggdonation • gamete bank • gamete don • gametes don • gametes sale • half sibling • oocyte bank • oocyte don • oocytes don
Sperm Donation	buying and selling gametes embryos • child donor • donate his sperm • donated gamete • donated sperm • donating sperm • donation of gamete • donor art • donor child • donor conceived offspring • donor conception • donor father • donor gamet • donor insemination • donor of gamet • donor of sperm or egg • donor offspring • donor semen • donor sibling • donorconceived child • gamete bank gamete don • gametes don • gametes sale • half siblings • known donors • semen bank • semen donor • single mother • sperm bank • sperm don • sperm from a known donor • spermdon
Embryo Donation	14 day limit • biobanking of eggs and embryo • buying and selling gametes embryos • donated eggs and embryo • donated embryo • donation embryo embryo adoption • embryo don • embryodon • embryos in excess • excess embryo • frozen embryo • surplus embryo
Surrogacy	commissioning parent • intended parent • shared gestation • subrog • surrog
Fertility Preservation	fp_ • cryopreserv • egg freez • embryo disposition • embryo freez • embryo storage • fertility preserv • fertilitypreserv • freezing egg • freezing oocyte • freezing ovari • frozen egg • frozen embryo • frozen sperm • not implanted embryo • oncofertility • oocyte preserv • ovarian tissue • post mortem • posthumous • postmortem • social freezing
Stem Cells	artificialgametes • embryo destructive research • embryo research • embryo scientific research • experimentation on embryos • gamete product • human embryo • oocytes for research • pluripotent • research on embryo • stem cell • totipot
Preimplantation Genetic Testing	_pgd_ • _pgs_ • _pgt_ • embryo select • enhancement • gender select • genetic counsel • genetic select • genetically select • hla matched • hla typing • pre implantation diagnos • pre implantatory exam • preimplantation diag • preimplantation or prenatal screening • preimplantational • preimplantatory • procreative benef • reproductive genetic • saviour sibling • selection of embryo • sex select • whole genome sequencing
Genetic Modification and Cloning	_ggm_ • chimer • clone • cloning • crispr • designer baby • enhancement • gametogene • gene edit • genome edit • genetic eng • genetic manipul • genetic modific • geneticeng • germ line • germline • nuclear transfer • posthuman • procreative benef • synthetic gamet
Mitochondrial Replacement Techniques	_mrt_ • mitochondrial disorder • mitochondrial gene trans • mitochondrial transfer • ooplasmic transfer • spindle transfer • three parent • tri parent
Assisted Insemination	_iui_ • insemination

*2267 articles were not assigned to any field and were labeled General (Gen)

## Appendix 2

Table 7

**Table 7:** Spearman’s correlations between ART fields and topics

	Correlation Coefficient
Egg Donation (S1) – Sperm Donation (S2)	0.521
Egg Donation (S1) – Family and Sociology (T5)	0.202
Sperm Donation (S2) – Assisted Insemination (S10)	0.337
Sperm Donation (S2)—Family and Sociology (T5)	0.341
Embryo Donation (S3) – Fertility Preservation (S5)	0.238
Surrogacy (S4) – Law and Religion(T4)	0.264
Fertility Preservation (S5) – Cost and Outcome (T2)	0.264
Fertility Preservation (S5) – Attitudes and Knowledge (T6)	0.264
Stem Cells (S6) – Genetic Modification (S8)	0.233
Stem Cells (S6) – Bioethics (T1)	0.343
PGT (S7) – Genetic Modification (S8)	0.213
PGT (S7) – Bioethics (T1)	0.265
Genetic Modification (S8) – Bioethics (T1)	0.413
Assisted Insemination (S10)—Family and Sociology (T5)	0.216

*For all pairs, p-value (2-tailed) = 0.000

## Appendix 3 Topic modeling output

Topic 1—Bioethics (green): ('0.027*"ethical" + 0.015*"genetic" + 0.014*"moral" + 0.008*"medical" + ''0.008*"legal" + 0.008*"ethic" + 0.007*"scientific" + 0.005*"principle" + ''0.005*"development" + 0.005*"medicine"').

Topic 2—Cost and Outcome (Bottom Red): ('0.040*"patient" + 0.019*"transfer" + 0.018*"rate" + 0.013*"cycle" + ''0.009*"clinical" + 0.009*"procedure" + 0.008*"risk" + 0.008*"clinic" + ''0.008*"cost" + 0.008*"ethical"').

Topic 3 – Law and Policy (Blue): ('0.024*"law" + 0.019*"legal" + 0.011*"country" + 0.010*"social" + ''0.009*"policy" + 0.008*"regulation" + 0.006*"legislation" + 0.006*"medical" '' + 0.006*"work" + 0.006*"analysis"').

Topic 4 – Psychology (Yellow): ('0.029*"couple" + 0.015*"psychological" + 0.015*"infertile" + ''0.010*"counselling" + 0.010*"factor" + 0.009*"patient" + ''0.009*"participant" + 0.008*"social" + 0.008*"relationship" + ''0.008*"interview"').

Topic 5 – Family and Sociology (Purple): ('0.092*"child" + 0.049*"parent" + 0.041*"family" + 0.022*"couple" + ''0.018*"mother" + 0.013*"lesbian" + 0.013*"conceive" + 0.012*"genetic" + ''0.012*"sex" + 0.011*"relationship"').

Topic 6 – Attitudes and Knowledge (Top Red) ('0.028*"patient" + 0.020*"age" + 0.013*"couple" + 0.013*"risk" + ''0.013*"attitude" + 0.012*"knowledge" + 0.009*"medical" + 0.008*"genetic" + ''0.007*"young" + 0.007*"participant"').

## Footnotes

1 When applying Latent Dirichlet Allocation (LDA) for Topic Modeling, shorter abstracts typically provide insufficient data to yield reliable results.

2 To lowerize and tokenize we used: https://tedboy.github.io/nlps/generated/generated/gensim.utils.simple_preprocess.html

3 We used a combination of open sources libraries including: nltk.corpus.stopwords.words('english') and https://github.com/seinecle/Stopwords/blob/master/src/main/java/net/clementlevallois/stopwords/resources/en.txt

4 First, we have formed bigram models using gensim open source library. Then, to lemmatized we use the open-source library spacy. (https://spacy.io/usage/models).

5 “gensim.corpora.Dictionary”.

6 I.e., transforming words/sentences into vectors (with 1 and 0): word: id mapping to apply TM afterwards [17. ].

7 https://radimrehurek.com/gensim/models/coherencemodel.html for TM using gensim.models.LdaMulticore with the following parameters: gensim.models.LdaMulticore(corpus = corpus, id2word = id2word, num_topics = k, random_state = 100, chunksize = 100, passes = 10, per_word_topics = True), k number of topics ranging from 2 to 15.

8 For example, “Portug” to capture Portugal and Portuguese, “Spain” and “Spani”, “Poland” and “Polish”. Also, cities names with double meaning (i.e., Male, Huntington) were carefully removed.

9 According to the United Nations Geoscheme: https://unstats.un.org/unsd/methodology/m49/ (some regions were merged due to proximity and low number of publications, i.e., Latin Americas and the Caribbean, Western and Southern Asia, Eastern Europe and Central Asia, Eastern and South-Eastern Asia).

10 According to the historical classification by the World Bank (per year of publication): https://datahelpdesk.worldbank.org/knowledgebase/articles/906519-world-bank-country-and-lending-groups

11 With a minimum cluster size = 3, and a minimum number of occurrences of a keyword = 20.

12 Russia was not among the 23 leading countries in the all period but only in the last years.
